# Statistics of Heat Transfer in Two-Dimensional Turbulent Rayleigh-Bénard Convection at Various Prandtl Number

**DOI:** 10.3390/e20080582

**Published:** 2018-08-07

**Authors:** Hui Yang, Yikun Wei, Zuchao Zhu, Huashu Dou, Yuehong Qian

**Affiliations:** 1 Faculty of Mechanical Engineering and Automation, Zhejiang Sci-Tech University, Hangzhou 310018, China; 2 State-Province Joint Engineering Lab of Fluid Transmission System Technology, Hangzhou 310018, China; 3 School of Mathematic Science, Soochow University, Suzhou 215006, China

**Keywords:** thermal dissipation rate, turbulence, Prandtl number, lattice Boltzmann method

## Abstract

Statistics of heat transfer in two-dimensional (2D) turbulent Rayleigh-Bénard (RB) convection for Pr=6,20,100 and $10^{6}$ are investigated using the lattice Boltzmann method (LBM). Our results reveal that the large scale circulation is gradually broken up into small scale structures plumes with the increase of Pr, the large scale circulation disappears with increasing Pr, and a great deal of smaller thermal plumes vertically rise and fall from the bottom to top walls. It is further indicated that vertical motion of various plumes gradually plays main role with increasing Pr. In addition, our analysis also shows that the thermal dissipation is distributed mainly in the position of high temperature gradient, the thermal dissipation rate $\varepsilon_{\theta }$ already increasingly plays a dominant position in the thermal transport, $\varepsilon_{u}$ can have no effect with increase of Pr. The kinematic viscosity dissipation rate and the thermal dissipation rate gradually decrease with increasing Pr. The energy spectrum significantly decreases with the increase of Pr. A scope of linear scaling arises in the second order velocity structure functions, the temperature structure function and mixed structure function(temperature-velocity). The value of linear scaling and the 2nd-order velocity decrease with increasing Pr, which is qualitatively consistent with the theoretical predictions.

## 1. Introduction

Thermal convection generally occurs in natural world and industrial field. Hartmann et al. 2001 [1] argued that for weather predictions the flow of atmosphere and thermal convection flow are not only related with smaller length scales and time scales, but also is closely greater scales for climate forecast. Marshall et al. (1999) [2] have investigated a key enforcing mechanical properties of ocean circulation in the ocean. Cardin and Olson (1994) [3] have studied that the enforced convection arises in the earth outer core. In general, the effect of rotation, the changing phases, complex boundary conditions and nonlinear dynamic can play a main role in many thermal convections. Lohse and Xia (2010) [4] and Chilla and Schumacher (2012) [5] respectively introduced research processes for turbulent RB convection. Turbulent RB convection is the most common phenomenon in natural convection [6]. Scheel et al. [7] presented the direct numerical calculations in a cylinder, which mainly focuses on the optimal scales of turbulent convective, especially, the analysis of the kinetic energy rates and thermal dissipation rates in the whole cell [7]. Hu [8] argued that the critical Ra (Rayleigh number) decreases for the beginning of convection, and the aspect ratio effects the critical value with increasing density inversion parameter in RB convection. Zhang [9] have studied the topic of the buoyancy ratio enhancing the fluid stability, and the critical Ra also gradually enhances with increasing value of the buoyant force ratio in thermal convection. Turbulent RB convection [10,11,12,13,14,15,16,17,18,19] has been studied numerically, experimentally and analytically. Shishkina [15] reported that massive thermal plumes rising in turbulent RB convection are analysed on account of 3D numerical calculations, which indicates that the thermal boundary layers are crucial to the temperature fields and velocity fields.

In RB convection, the Ra (a measure of the buoyant force), and the Prandtl number Pr=ν/κ (the kinematic viscosity divides the thermal diffusivity) are imporpant dimensionless numbers. Krishnamurti [20,21] performed extensive convection experiments on mercury (Pr∼0.02), air (Pr∼0.7), water (Pr∼6.8), freon (Pr∼7), and silicon oil (Pr∼100). Busse and Whitehead [22] also reported the jagged instability and cross-roll instability of silicon oil in experiment, which indicated that the results of two-dimensional (2D) convection are closely similar to that of 3D convection for large Pr convection [10,11].

This survey to investigate the bifurcation and chaos in convection with large Pr and different Pr using a low-dimensional model containing is exploited in this paper. The main purpose of our paper is to offer ‘reference’ cases for comparison with a host of various flows. In general, the laboratory experiments are performed by using fluids with Pr up to $10^{4}$ behave. However, a similar flow for infinite Pr does not exist. Although a model of a plume is not investigated in the Earth’s mantle, the flow possessing overwhelmingly large Pr is close to the mantle investigations since the mantle possesses a Pr of $10^{6}$. Our results mainly appraise the variety of physical mechanism phenomenon about the kinematic viscosity dissipation, thermal dissipation, energy spectra, temperature spectra and the 2nd structure function with increasing Pr at the same Ra. Our studies mainly focus on the characteristics of the above physical mechanism phenomena with increasing Pr, the characteristics of flow and scale properties in turbulent RB system at large Prandtl number. In addition, the Bolgiano-Obukhov-like (BO59) scaling is used to explore the profound insight of the velocity fluctuation and the temperature fluctuation with the increase of Pr in turbulent RB convection.

The lattice Boltzmann scheme is performed to simulate in all numerical examples. The lattice Boltzmann method (LBM) had possessed latent advantages in previous studies to multiphase, single, heat and mass transfer hydrodynamic problems [23,24,25,26,27,28,29,30,31,32,33,34,35,36]. The LBM is particularly propitious to attack complex boundary conditions. It is a attractive topic that the turbulent flows are simulated by the LBM [28]. The LBM on account of Direct Numerical Simulations (DNS), Large Eddy Simulations (LES) also possess great success in turbulence predictions [34]. In general, in turbulent RB convection, Rayleigh number (Ra) is a very important dimensionless parameter. When Ra is less than $10^{6}$, the flow state is laminar RB convection. When Ra is between $10^{6}$ and $10^{9}$, the flow state is soft turbulent RB convection. However, when Ra is greater than $10^{9}$, the flow state is full turbulent RB convection.

The main structure of the paper is introduced. Firstly, a thermal lattice Boltzmann model is introduced. Secondly, the numerical verifications are presented. Thirdly, the result analysis and discussions are provided. Finally, some conclusions are provided.

## 2. Mathematical Equation of Fluid and Lattice Boltzmann Method

The discrete kinetic model is based on LBM. A double-population approach using the lattice Boltzmann equation is proposed by Shan [35]. The lattice Boltzmann equation of computing dynamics and the lattice Boltzmann equation of computing advection equation are described by the following expressions, correspondingly [26,32]:

(1) $$f_{i}\left(x_{\alpha }+c_{i\alpha }\delta t,t+\delta t\right)-f_{i}\left(x_{\alpha },t\right)=-\omega_{1}\left[f_{i}\left(x_{\alpha },t\right)-f_{i}^{\left(eq\right)}\left(x_{\alpha },t\right)\right]+F_{i}$$

(2) $$g_{i}\left(x_{\alpha }+c_{i\alpha }\delta t,t+\delta t\right)-g_{i}\left(x_{\alpha },t\right)=-\omega_{2}\left[g_{i}\left(x_{\alpha },t\right)-g_{i}^{\left(eq\right)}\left(x_{\alpha },t\right)\right]$$

where $f_{i}$, $g_{i}$ denote the probability density functions at (x,t), $c_{i}$ belongs to a discrete velocity, $F_{i}$ is the mesoscopic buoyant body force, $\omega_{1}$ is the flow relaxation time and $\omega_{2}$ is the temperature relaxation time in the above equations, respectively. The local kinetic equilibrium $f_{i}^{eq}$ is equilibrium function for flow, and $g_{i}^{eq}$ is the equilibrium function for temperature [32].

(3) $$f_{i}^{eq}\left(x_{\alpha },t\right)=w_{i}\rho \left\{1+\frac{c_{i\alpha }u_{\alpha }}{c_{s}^{2}}+\frac{u_{\alpha }u_{\beta }}{2c_{s}^{2}}\left(\frac{c_{i\alpha }c_{i\beta }}{c_{s}^{2}}-\delta_{\alpha \beta }\right)\right\}$$

(4) $$g_{i}^{eq}\left(x_{\alpha },t\right)=w_{i}\theta \left\{1+\frac{c_{i\alpha }u_{\alpha }}{c_{s}^{2}}+\frac{u_{\alpha }u_{\beta }}{2c_{s}^{2}}\left(\frac{c_{i\alpha }c_{i\beta }}{c_{s}^{2}}-\delta_{\alpha \beta }\right)\right\}$$

where $c_{s}$ is the sound speed and $w_{i}$ denotes the weight coefficients.

(5) $$c_{s}=\frac{1}{\sqrt{3}}\frac{\delta x}{\delta t},\;w_{i}=\left\{\begin{array}{cc} \frac{4}{9} & i=0\\ \frac{1}{9} & i=1∼4\\ \frac{1}{36} & i=5∼8 \end{array}\right.$$

The relation between the relaxation parameter $\omega_{1}$ and the kinematic viscosity ν is presented and the relaxation is also presented between the parameter $\omega_{2}$ and the fluid thermal diffusivity κ.

(6) $$\nu =c_{s}^{2}\left(\frac{1}{\omega_{1}}-\frac{1}{2}\right)\delta t,\;\kappa =c_{s}^{2}\left(\frac{1}{\omega_{2}}-\frac{1}{2}\right)\delta t$$

The mesoscopic buoyant body force with the Boussinesq approximation is formulated by the following equation

(7) $$F_{i}=3w_{i}\rho g\theta \beta c_{i\alpha }$$

in which *g* denotes the gravity acceleration, β denotes the coefficient of thermal expansion, and $c_{i\alpha }$ denotes the *y*-component of $c_{i}$ correspondingly.

The relations are defined as coarse-grained (in velocity space) fields of the distribution functions for the macroscopical density, momentum, and temperature, which are given by

(8) $$\rho =\sum_{i}f_{i},\;\rho u_{\alpha }=\sum_{i}f_{i}c_{i\alpha },\;\theta =\sum_{i}g_{i}$$

The Equations (1) and (2) are respectively expansed by a Chapman-Enskogm approach, which leads to the macroscopical equations of fluid. The inertial terms are reproduced by the streaming step on the left-hand side in the hydrodynamical equations, whereas the kinematic viscosity coefficient and the coefficient of thermal diffusion are respectively connected to the relaxation (towards equilibrium) properties in the right hand side.

(9) $$\partial_{t}\rho +\partial_{\alpha }\left(\rho u_{\alpha }\right)=0$$

(10) $$\partial_{t}\left(\rho u_{\alpha }\right)+\partial_{\beta }\left(\rho u_{\alpha }u\beta \right)=-\partial_{\alpha }\left(P\right)+\partial_{\beta }\left(2\rho \nu S_{\alpha \beta }\right)-g\beta \Delta \theta$$

(11) $$\partial_{t}\left(\theta \right)+u_{\alpha }\partial_{\alpha }\left(\theta \right)=\kappa \partial_{\alpha }\partial_{\beta }\left(\theta \right)$$

The Ra is an important variable in the natural convection. The definition of Ra is

(12) $$Ra=\frac{\beta \Delta \theta gH^{3}}{\nu \kappa }$$

in which ν is the kinematic viscosity, κ is the thermal diffusivity and β is the coefficient of isobaric thermal expansion, Δθ is the temperature difference between the bottom boundary and top boundary in cavity, *g* is the gravity acceleration, and *H* is the height of cavity.

The perturbed is given in the initial stage of simulation. Once RB convection is taken shape, the heat transfer near wall is overwhelmingly strengthened. The Nusselt number denotes the enhancement of the heat transfer. The expression of Nu is presented in our simulations.

(13) $$Nu=1+\frac{<u_{y}\theta >}{\left(\kappa \Delta \theta /H\right)}$$

in which $u_{y}$ denotes the vertical velocity and <> is the spatial average in the whole flow domain.

## 3. Calculation Results and Discussions

In the present study, numerical calculations in 2D turbulent RB convection for Pr=6,20,100 and $10^{6}$ are carried out at $Ra=5.4\times 10^{9}$. The computing grid is set to 2012×2012. The no-slip boundary condition is performed on top boundary and on the bottom boundary. And left and right boundary conditions are also performed by the no-slip boundary condition in all numerical calculations. The initial dimensionless temperature in bottom boundary is 1, and the initial dimensionless temperature of top boundary is 0, respectively. Meanwhile, a linear distribution of the dimensionless temperature from 0 to 1 is performed between top boundary and bottom boundary.

**Figure 1:**
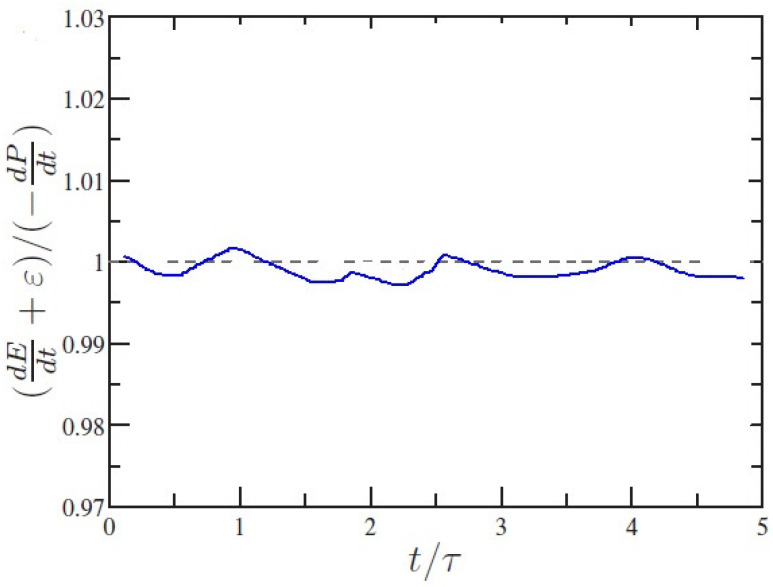
Validation of the energy balance relation (14) for the LBM scheme.

The budget relation of instantaneous kinetic energy is used to test and verify the accuracy of the numerical calculations.

(14) $$-\frac{dP(t)}{dt}=\frac{dE(t)}{dt}+\varepsilon \left(t\right)$$

where P(t)=−βg∫∫yθ(x,y,t)dxdy is the total potential energy, $E\left(t\right)=\;\int \int \;0.5[u{\left(x,y,t\right)}^{2}+v{\left(x,y,t\right)}^{2}]dxdy$ denotes the total kinetic energy and $\varepsilon \left(t\right)=\;\int \int \;\nu {\left[\partial_{i}u_{j}\left(x,y,t\right)\right]}^{2}dxdy$ denotes the dissipation rate of total kinetic energy. The ratio of the right-hand side to the left-hand side of Equation (14) as a relation of the normalized time t/τ is presented in Figure 1. One can see that the difference within only 0.6% at all times is achieved at the energy balance equation, which is indicated that the accuracy of LBM is reliable.

### 3.1. Global Quantities of Turbulent RB Convection

**Figure 2:**
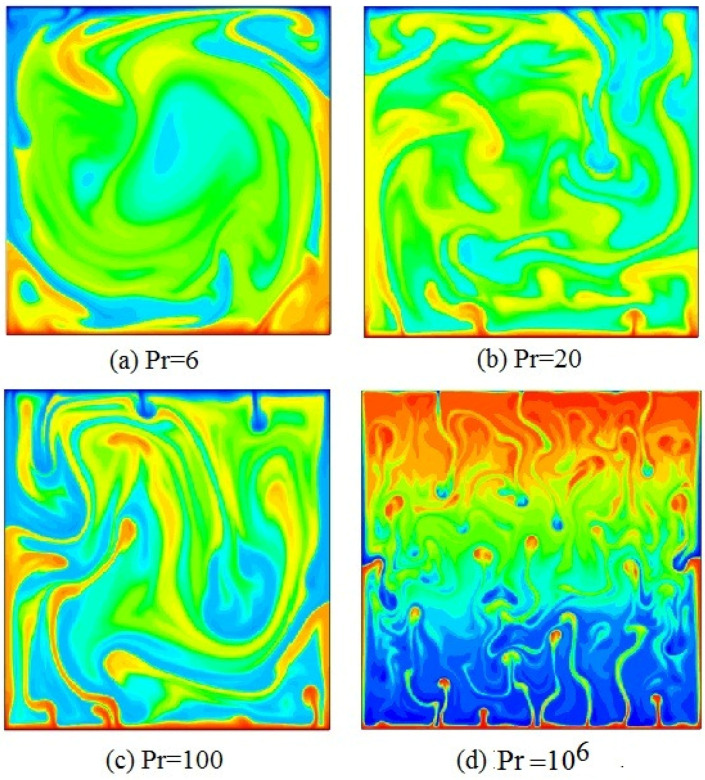
Temperature distributions with steady flow at Pr=6,20,100, $10^{6}$ (**a**–**d**), and $Ra=5.4\times 10^{9}$.

In Figure 2, the temperature distribution with superimposed temperature fields at Pr=6,20,100, $10^{6}$ (from a to d), and $Ra=5.4\times 10^{9}$ is displayed for several elective coarseness types, where is the flow characteristic of RB convection system, the blue regions denote cold fluid, and the red regions correspond to hot fluid. One can see that large-scale circulations are obtained in the center of the cavity at Pr=6. A large number of thermal plumes are predominantly rising in corners of the cavity, and the cold plumes are falling in corners of the cavity, which can be main reason owing to a large scale circulation that is guided along one of diagonals of the cavity. It is demonstrated that large-scale circulations of the fluid in cavity are decomposed gradually with the increase of Pr, which is consistent with the experiment for large Pr [4]. It is also showed that vertical motion of various plumes gradually plays main role with increasing Pr. Respectively, a large number of small-scale thermal plumes vertically rise from the bottom wall to top wall, a large number of small-scale cold plumes vertically fall from the top boundary to the bottom boundary, and a great number of small eddies appear at $Pr=10^{6}$. The longitudinal velocity play a predominant role in cavity with increasing Pr. It is further found that the average time of achieving steady-state increase overwhelmingly with increasing Pr.

In the following section, Grossmann -Lohse theory for is used to investigate the dissipation rate of kinetic-energy rate and the thermal dissipation rate, which is the following equations [6]

(15) $$\varepsilon_{u}\left(t\right)={\langle \nu {\left[\partial u_{i}\left(x,y,t\right)\right]}^{2}\rangle }_{V}=\frac{\nu^{3}}{L^{4}}\left(Nu-1\right)RaPr^{-2}$$

(16) $$\varepsilon_{\theta }\left(t\right)={\langle \kappa {\left[\partial \theta_{i}\left(x,y,t\right)\right]}^{2}\rangle }_{V}=\kappa \frac{\Delta^{2}\theta }{L^{2}}Nu$$

The relations of Equations (15) and (16) are able to be derived from the Boussinesq equations [6].

**Figure 3:**
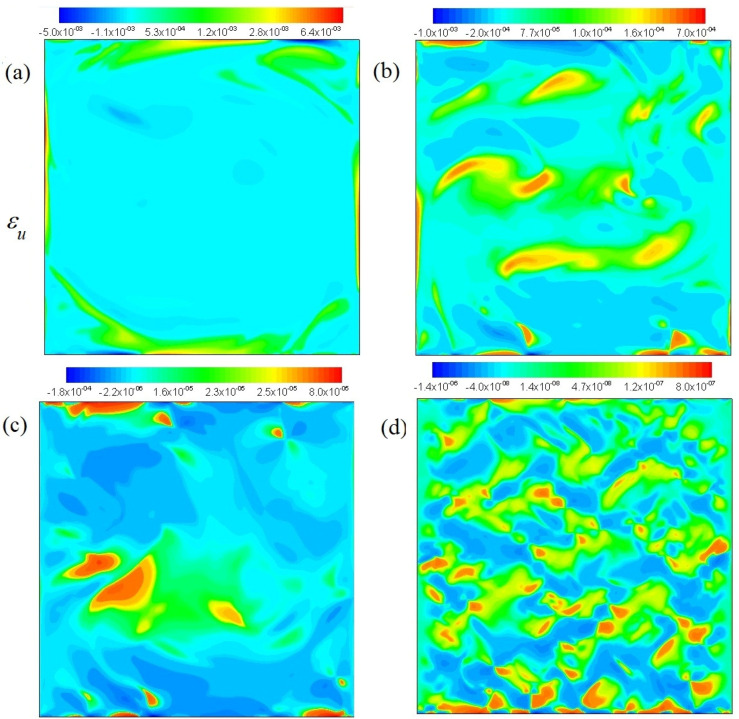
Kinetic energy dissipation rates Pr=6,20,100, $10^{6}$ (**a**–**d**), and $Ra=5.4\times 10^{9}$.

The distributions of kinetic-energy dissipation rate at Pr=6,20,100, $10^{6}$ (from a to d), and $Ra\;=\;5.4\times 10^{9}$ are shown in Figure 3. It is shown that the value of kinetic-energy dissipation rate decreases with the increase of Pr in cavity. The kinetic-energy dissipation rate at Pr=6 plays a major role around cavity. The kinetic-energy dissipation rate is occupied gradually in the interior of cavity with increasing Pr. The kinetic-energy dissipation rate becomes gradually dispersed at $Pr=10^{6}$.

**Figure 4:**
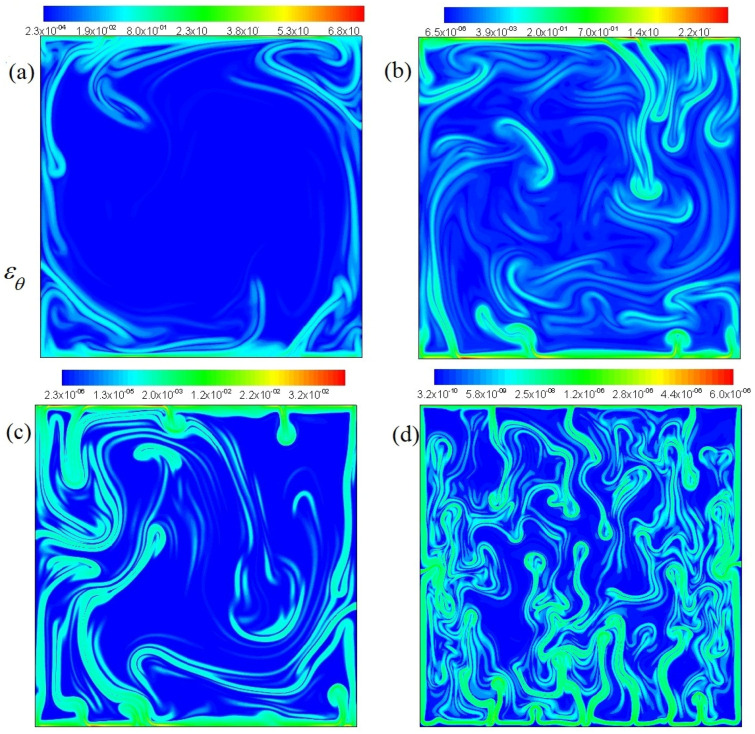
Thermal energy dissipation rates at Pr=6,20,100, $10^{6}$ (**a**–**d**), and $Ra=5.4\times 10^{9}$.

The distributions of thermal dissipation rate at Pr=6,20,100, $10^{6}$ (from a to d) are shown in Figure 4, respectively. One can see that the value of thermal dissipation rate also decreases with the increase of Pr in cavity. The thermal dissipation rate at Pr=6 plays a major role around cavity, similarly. The thermal dissipation rate gradually expands in the interior of cavity with increasing Pr. A large number of thermal dissipation emerge in the whole domain at $Pr=10^{6}$. From a detailed comparison of Figure 3 with Figure 4, we can see that the thermal dissipation is distributed mainly in the position of high temperature gradient. In other word, the intense thermal dissipation events mainly concentrate on the interfaces of hot and cold fluids and these interfaces between the hot fluid and the cold fluid develop to complex associate structures with large intricate.

**Figure 5:**
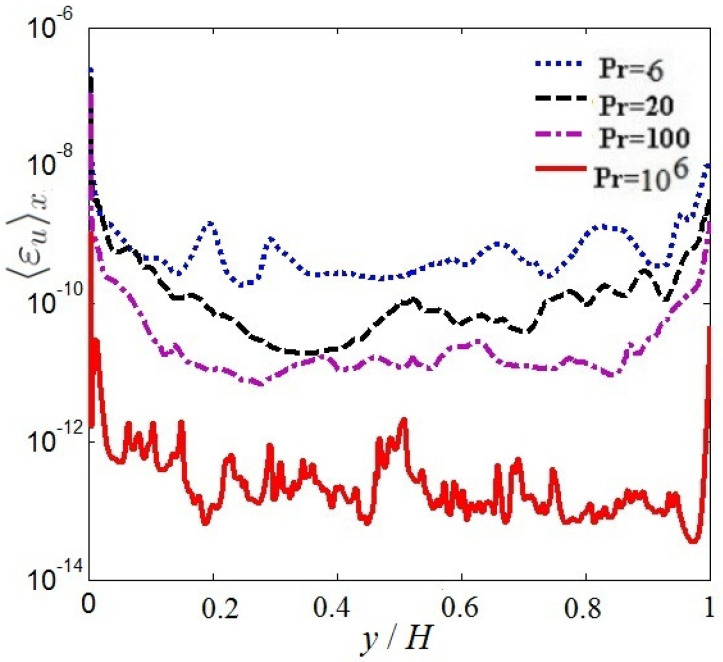
Mean vertical kinetic-energy dissipation rate profiles.

**Figure 6:**
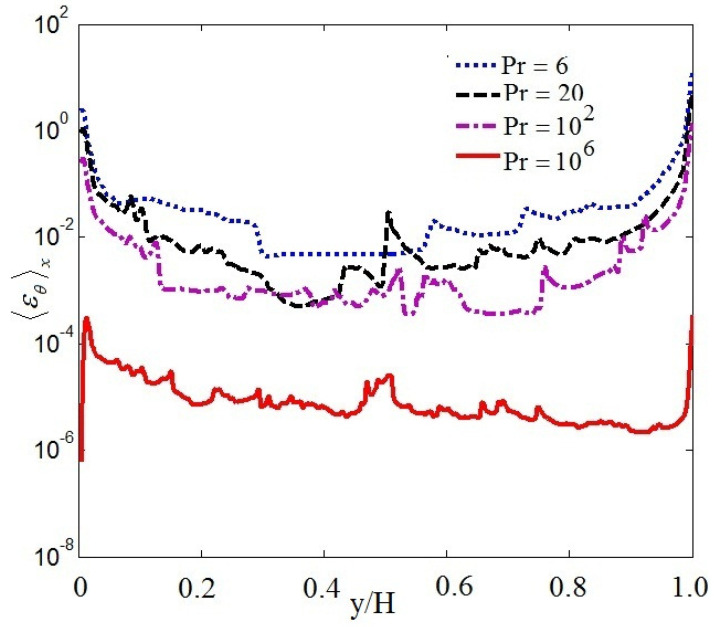
Mean vertical thermal dissipation rate profiles.

Figure 5 and Figure 6 display the mean vertical dissipation rate profiles of kinetic-energy ${\langle \varepsilon_{u}\rangle }_{x}$ and the mean vertical thermal dissipation rate profiles ${\langle \varepsilon_{\theta }\rangle }_{x}$ for Pr=6,20,100, $10^{6}$, respectively, where ${\langle \ldots \rangle }_{x}$ represents a horizontal average. As observed in Figure 5 and Figure 6, the mean vertical dissipation rate profiles of kinetic-energy and the mean vertical thermal dissipation rate profiles significantly decrease with increasing Pr. The strong dissipation events mainly arises near the top and bottom boundary in the turbulent range. It is further seen that the thermal dissipation rate $\varepsilon_{\theta }$ already plays a key role in the thermal energy transport, thus $\varepsilon_{u}$ may be neglected in virtue of inverse cascade of kinetic energy in thermal convection.

**Figure 7:**
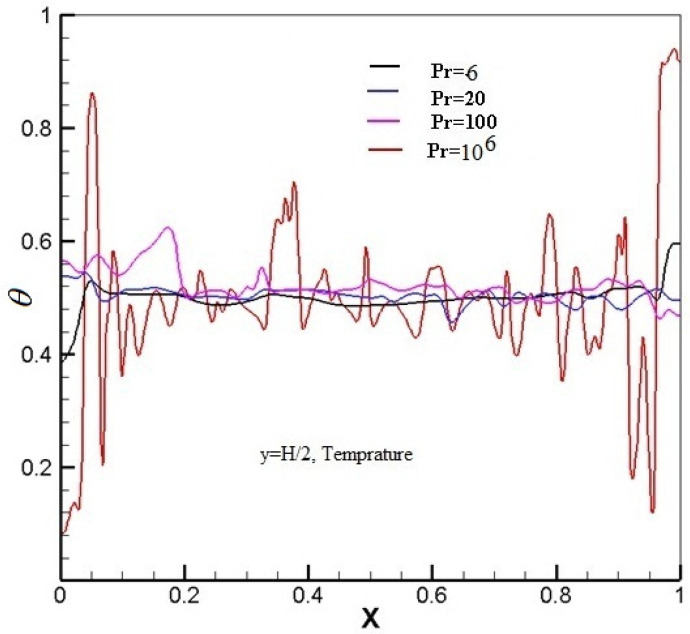
Instantaneous temperature distributions with superimposed temperature fields at Pr = 6, 20, 100, $10^{6}$, and $Ra=5.4\times 10^{9}$.

The contrast of the temperature on the midline (y = H/2) at Pr=6,20,100, $10^{6}$, and $Ra\;=\;5.4\times \;10^{9}$ is displayed in Figure 7. Plotted in Figure 7, it is noted that the relative magnitude of temperature difference is increased overwhelmingly with increasing Pr, and the variety of temperature is also enhanced drastically.

The Bolgiano-Obukhov scaling is used to judge the dissipation scale in 2D turbulence thermal convection. The rigorous relations are obtained by assuming spatial homogeneity, within the Boussinesq approximations equation [4].

(17) $${\langle \varepsilon_{u}\rangle }_{V}=\frac{Nu-1}{\sqrt{RaPr}}$$

(18) $${\langle \varepsilon_{\theta }\rangle }_{V}=\frac{Nu}{\sqrt{RaPr}}$$

**Figure 8:**
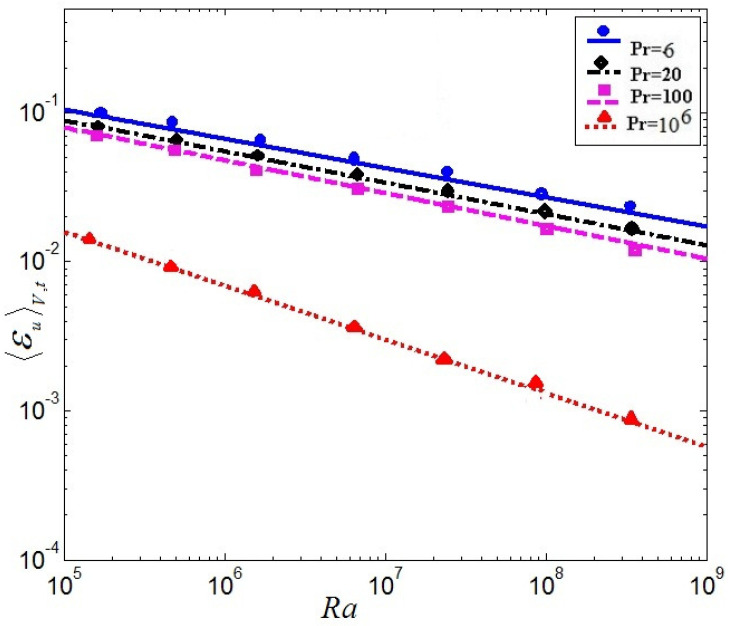
Volume and time-averaged kinetic dissipation rates versus the Rayleigh number Ra.

**Figure 9:**
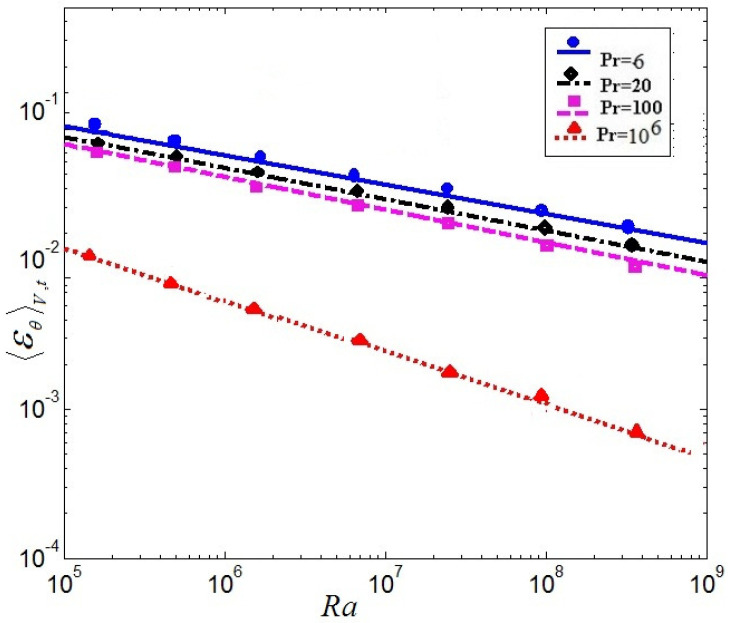
Volume and time-averaged thermal dissipation rates versus the Rayleigh number Ra.

**Figure 10:**
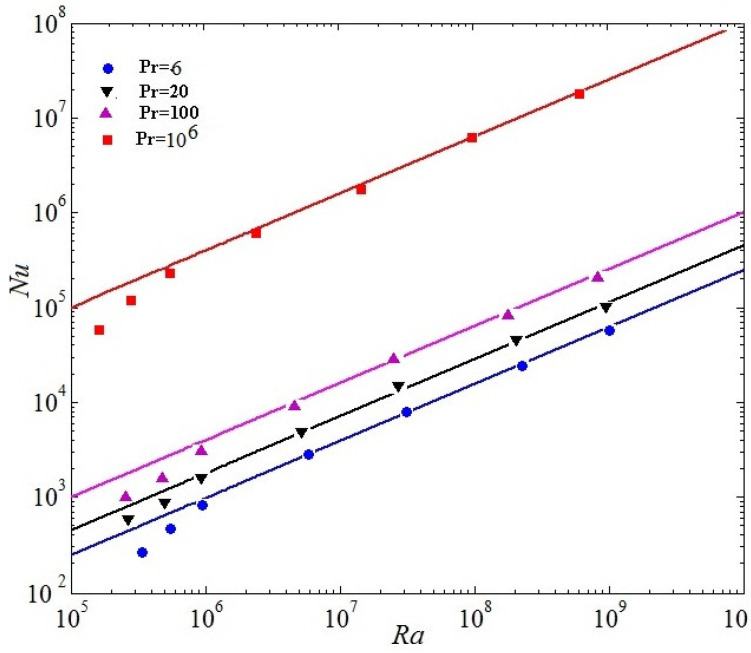
Nusselt number as a function of Rayleigh number at Pr=6,20,100, and $10^{6}$.

The time and volume -averaged kinetic dissipation rates versus the Rayleigh number Ra, the time and volume-averaged thermal dissipation rates versus the Ra and Nusselt number Nu as a function of Ra at Pr = 6, 20, 100, and $10^{6}$ between numerical results and theoretical values are given in Figure 8, Figure 9 and Figure 10. Figure 8 represents the so-called volume and time-averaged kinetic dissipation rates versus the Rayleigh number Ra. Figure 9 represents the thermal dissipation rates versus the Ra in the so-called volume and time-averaged. The solid lines represent the theoretical relation as relations (17) and (18). The dispersed points are the results of the present LBM. It is mainly demonstrated that the numerical calculation results of the LBM are excellently consistent with the theoretical value for time-averaged kinetic dissipation rates and thermal dissipation rates versus the Ra in Figure 8 and Figure 9.

**Figure 11:**
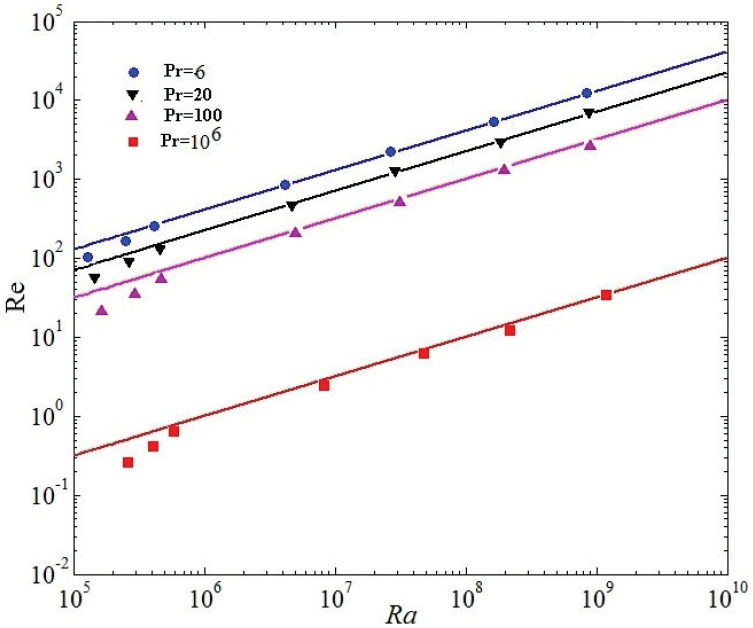
Reynolds number as a function of Rayleigh number at Pr=6,20,100, and $10^{6}$.

The relation of turbulent heat flux and the relation of the kinetic energy will be discussed, respectively. Figure 10 demonstrates the Nusselt number as a relation of Rayleigh number at Pr=6,20,100, and $10^{6}$. Figure 11 manifests the Reynolds number (Re) as a relation of Rayleigh number, respectively. Nusselt is wall averaged Nusselt number in the whole computational domain. The Grossmann-Lohse theory had offered a good insight for the of comprehension of Nu(Ra,Pr), Re(Ra,Pr) (See Equations (19) and (20)) and even allowed multifarious predictions where the bulk turbulence plays an important role both the holistic kinetic dissipation and the system thermal dissipation [6].

(19) $$Nu∼Ra^{0.5}Pr^{0.5}$$

(20) $$Re∼Ra^{0.5}Pr^{-0.5}$$

The relations (19) and (20) respectively represent the steady state regime of RB turbulence, which was proposed for turbulent RB convection at overwhelmingly high Ra by Kraichnan, and then developed by Grossmann and Lohse in their Pr−Ra relations [6]. The ultimate state scaling was observed numerically [37] and experimentally [38,39], where the ultimate regime scaling is achieved when the solid boundaries are lacked. Plotted in Figure 10 and Figure 11, it is noted that a linear scaling can be presented for for both Nu(Ra) and Re(Ra) for nearly four decades from Ra $10^{6}$ to $10^{10}$. It is validated that the energy spectrum significantly decreases with the increase of the Pr. Our results further demonstrate that the numerical calculation results of the LBM are well consistent with the Grossmann-Lohse relations for Nu(Pr,Ra) and Ra(Pr,Ra) [6] from Ra = $10^{6}$ to $10^{10}$ in Figure 10 and Figure 11.

### 3.2. Scaling of Energy Spectra, Fluxes and Spatial Intermittency

**Figure 12:**
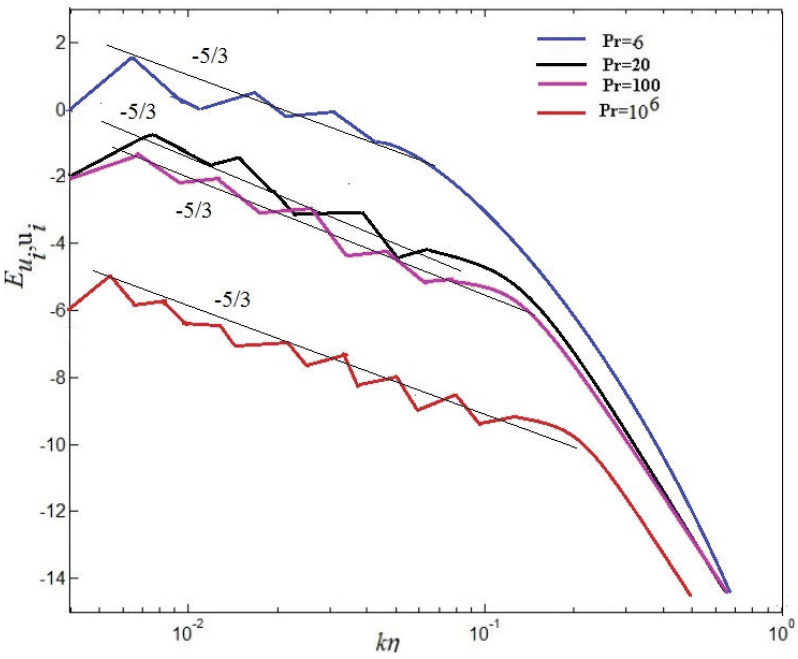
Kinetic energy spectra at different Pr.

**Figure 13:**
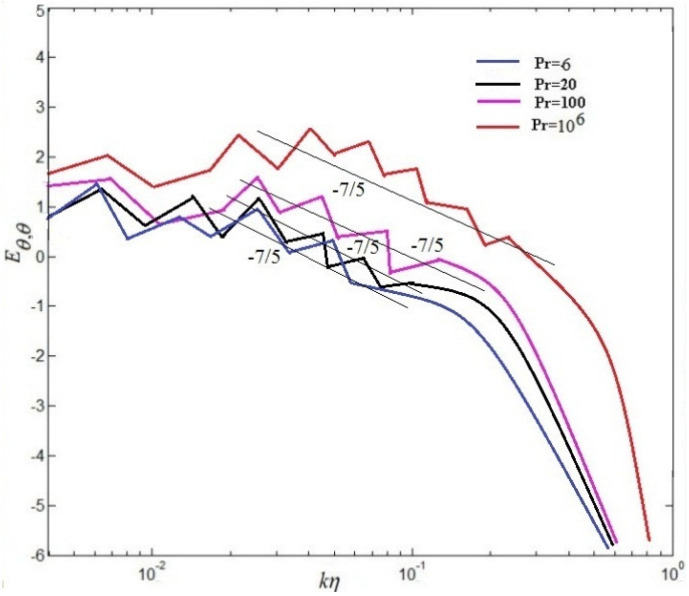
Temperature spectra at different Pr.

The scaling of energy spectra and fluxes will be discussed in the following section. For passive scalars, the value 5/3 is in agreement with Obukhov-Corrsin scaling [6], and consistent with experimental findings of Kolmogorov scaling along the center line of turbulent convection [9]. Temperature power spectra have been investigated previously by some researchers [37,38].

(21) $$E_{u}\left(k\right)∼\varepsilon^{\frac{2}{3}}k^{-\frac{5}{3}}F\left(\eta k\right)$$

and

(22) $$E_{\theta }\left(k\right)∼{\left(\beta g\right)}^{-\frac{2}{5}}\varepsilon_{\theta }^{\frac{4}{5}}k^{-\frac{7}{5}}$$

in which F(.) denotes a single dimensionless relation of a dimensionless argument. The kinetic spectra and temperature spectra in central point(Nx/2,Ny/2) at time scales are showed in Figure 12 and Figure 13, respectively. These results demonstrate that the energy spectrum is significantly decreased by the increase of the Pr in Figure 12. For the dimensionless inertial range (0.03<kη<0.05), the numerical results are consistent with Kolmogorov’s 5/3 spectrum at Pr=6. For Pr=20, the numerical results well agree with Kolmogorov’s 5/3 spectrum at the dimensionless inertial range (0.04<kη<0.1). For the dimensionless inertial range (0.04<kη<0.16), the numerical results are consistent with Kolmogorov’s 5/3 spectrum at Pr=100. For $Pr=10^{6}$, excellent agreement between the numerical results and Kolmogorov’s 5/3 spectrum is presented at the dimensionless inertial range (0.06<kη<0.21). It is found that the dimensionless inertial range is enlarged with increasing Pr, which is demonstrated that the energy dissipation rate scaling rises ($\epsilon_{u}∼\nu^{3}Re^{3}/L^{4}$) and the thermal dissipation rate ($\epsilon_{\theta }∼\kappa \Delta \theta^{2}RePr/L^{2}$) increases gradually with increasing Pr. It is also demonstrated that current results could be explained near 7/5 at low and near 5/3 at lightly higher frequencies, respectively, agree with the collapse well on top of each other in the Bolgiano-Obukhov-like scaling [4,6]. To investigate the spatial intermittency effects of turbulent RB convection for different Prandtl numbers, the longitudinal velocity (See Equation (19))and the structure function of temperature (See Equation (20))over horizontal separations are regarded. The 2nd-order structure functions of velocity fluctuations and the 2nd-order structure functions of temperature fluctuations are hoped to next expressions, which are the most-studied quantities in buoyancy-driven turbulence [4].

(23) $$S_{2}\left(r,t\right)\equiv \langle |\delta u_{r}\left(t\right){{|}^{2}\rangle }_{V}\equiv \frac{\nu^{2}}{L^{4}}\left(Nu-1\right)RaPr^{-2}$$

It is found that tht value of linear scaling decreases with the increase of Pr.

(24) $$R_{2}\left(r,t\right)\equiv \langle |\delta \theta_{r}\left(t\right){{|}^{2}\rangle }_{V}\equiv \frac{{△}^{2}}{H^{2}}Nu$$

in which △ denotes the difference between the top wall and the bottom wall, and *H* denotes height of cavity. These relations can be easily derived from the Boussinesq equations and the corresponding boundary conditions [6]. Secondly, the mixed velocity-temperature structure function is expected to following expression

(25) $$M\left(r,t\right)\equiv \langle |\delta u_{r}\left(t\right)\delta \theta_{r}\left(t\right){|\rangle }_{V}\;$$

**Figure 14:**
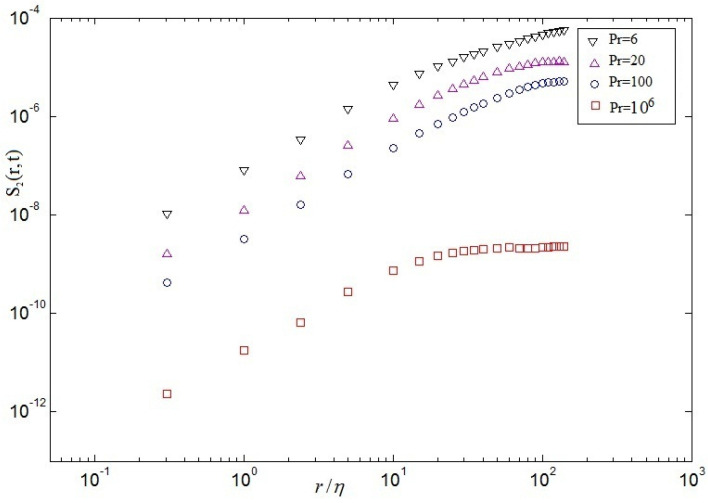
2nd-order velocity structure functions at different Pr.

**Figure 15:**
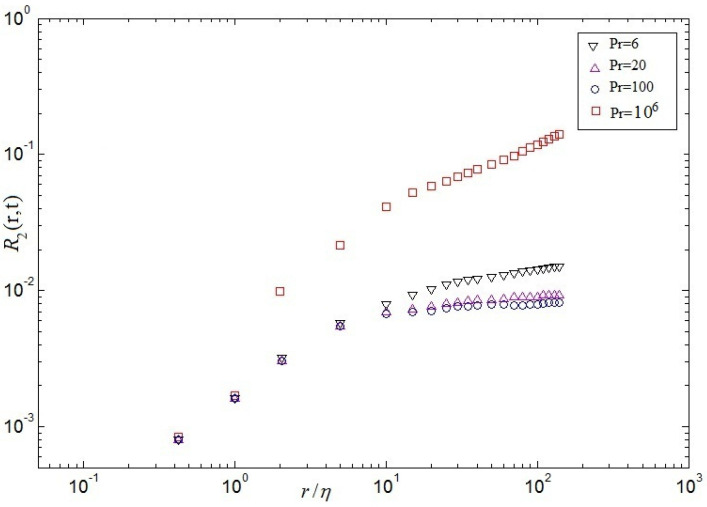
2nd-order temperature structure functions at different Pr.

**Figure 16:**
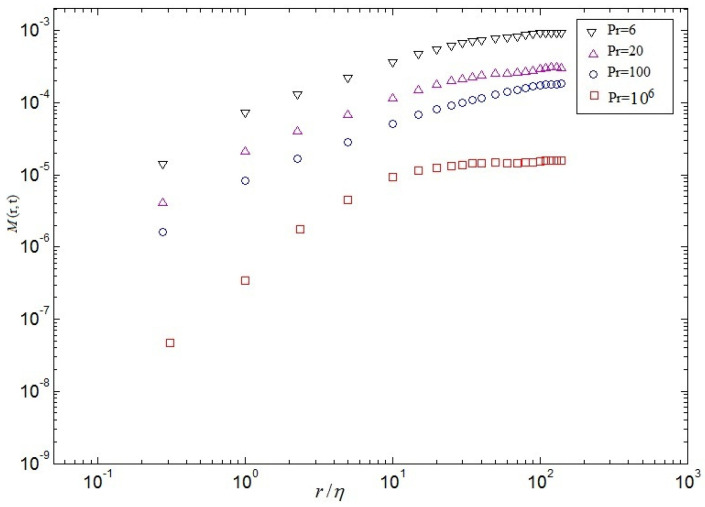
2nd-order mixed velocity-temperature structure function at different Pr.

Figure 14, Figure 15 and Figure 16 demonstrate respectively the second order structure function of velocity $S_{2}\left(r\right)$, structure function of temperature $R_{2}\left(r\right)$ and the mixed structure function (velocity-temperature) M(r) at the whole computational domain computed at a late stage of the self-similar regime. As can be seen from Figure 14, as the Pr increases, the 2nd-order velocity decreases, which is qualitatively consistent with the theoretical predictions. It is validated that the 2nd-order velocity structure functions display a series of linear scaling that extends to larger scales for different Prandtl numbers, i.e, 5≤r/η≤50 for Pr=6, 1≤r/η≤35 for Pr=20, 1≤r/η≤30 for Pr=100 and 0.1≤r/η≤10 for $Pr=10^{6}$ in Figure 14. It is validated that the value of linear scaling decreases with the increase of Pr, which may be given rise to large-scale circulation disappearing, the secondary eddies arising, and the dissipation of energy playing a major role at $Pr=10^{6}$ in cavity, respectively. Noting that as the Pr increases, the 2nd-order velocity decreases, which is qualitatively consistent with the theoretical predictions (See Equation (19)). It is also demonstrated that a range of linear scaling emerges in the 2nd-order structure functions of velocity fluctuations, the 2nd-order structure functions fluctuations of temperature and the 2nd-order structure functions of mixed structure function, which may be in virtue of the small-scale temperature structures, like the thermal spikes or plumes that possess strong corrections. Plotted in Figure 15 and Figure 16, the 2nd-order structure functions of temperature and the mixed structure function are also qualitative in agreement with those observed in Figure 14.

## 4. Conclusions

Numerical calculations of two-dimensional turbulent RB convection at large Prandtl number are investigated using LBM. Our results demonstrate that the variation characteristics of physical mechanism phenomenon about the kinematic viscosity dissipation, thermal dissipation, energy spectra, temperature spectra and the 2nd structure function with increasing Pr at the same Ra. The main conclusions are as follows:

Fisrtly, the large scale circulation in cavity is gradually broken up into small scale structures that thermal plumes rise and fall from the bottom to top walls with increasing Pr, which agrees with the visualization of the experiment. The large-scale circulations of the fluid are decomposed gradually with the increase of Pr. It is found that the vertical motion of various plumes gradually play a main role with increasing Pr. Especially, a large number of small-scale thermal plumes vertically rise from the bottom wall to top wall, a large number of small-scale cold plumes vertically fall from the top wall to the bottom wall, and a great deal of small eddies appear at $Pr=10^{6}$. Secondly, the kinematic viscosity dissipation rate and the thermal dissipation rate gradually decrease with increasing Pr, which is qualitatively consistent with the theoretical predictions. The intense thermal dissipation events mainly concentrate on the interfaces of hot fluid and cold fluid. The thermal dissipation rate $\varepsilon_{\theta }$ already plays a key role in the thermal energy transport, thus $\varepsilon_{u}$ can have no effect in virtue of inverse cascade of kinetic energy in thermal convection. It is also demonstrated that the numerical calculation results of the LBM are excellently consistent with the theoretical value for time-averaged kinetic and thermal dissipation rates versus the Ra. In addition, the energy spectrum significantly decreases with increasing Pr, which indicates that the numerical results of the LBM agree with the Grossmann-Lohse theory for Nu(Pr,Ra) and Ra(Pr,Ra) [6] from Ra $10^{6}$ to $10^{10}$. Finally, the 2nd-order velocity decreases with increasing Pr, which is qualitatively consistent with the theoretical value. It is also found that a scope of linear scaling appears in the second order structure functions of velocity fluctuations, the second order structure functions of temperature fluctuations and mixed structure function, which may be in virtue of the small-scale temperature structures, like the thermal plumes or spikes.

Due to natural parallelism of the LBM, the LBM may have potential prospects to simulate turbulent RB convection. Even though the results of two-dimensional cases are reported in this paper, similar scaling laws may appear (the Kolmogorov-Obukhov scenario) in 3D and it might be crucial to modify parametric assembles in numerical simulations. Accordingly, allowed us to evaluate the importance of the Kolmogorov-Obukhov scenario in three dimensions at large Pr. Our results discussed in this paper provide insights into the flow dynamics of RB convection. These results will be useful in modelling convective flows in the atmospheres and interiors of stars and planets, as well as in the applications of engineer.
